# Screening SIRT1 Activators from Medicinal Plants as Bioactive Compounds against Oxidative Damage in Mitochondrial Function

**DOI:** 10.1155/2016/4206392

**Published:** 2016-02-11

**Authors:** Yi Wang, Xinying Liang, Yaqi Chen, Xiaoping Zhao

**Affiliations:** ^1^College of Pharmaceutical Sciences, Zhejiang University, Hangzhou 310058, China; ^2^College of Preclinical Medicine, Zhejiang Chinese Medical University, Hangzhou 310053, China

## Abstract

Sirtuin type 1 (SIRT1) belongs to the family of NAD^+^ dependent histone deacetylases and plays a critical role in cellular metabolism and response to oxidative stress. Traditional Chinese medicines (TCMs), as an important part of natural products, have been reported to exert protective effect against oxidative stress in mitochondria. In this study, we screened SIRT1 activators from TCMs and investigated their activities against mitochondrial damage. 19 activators were found in total by* in vitro* SIRT1 activity assay. Among those active compounds, four compounds, ginsenoside Rb_2_, ginsenoside F1, ginsenoside Rc, and schisandrin A, were further studied to validate the SIRT1-activation effects by liquid chromatography-mass spectrometry and confirm their activities against oxidative damage in H9c2 cardiomyocytes exposed to tert-butyl hydroperoxide (t-BHP). The results showed that those compounds enhanced the deacetylated activity of SIRT1, increased ATP content, and inhibited intracellular ROS formation as well as regulating the activity of Mn-SOD. These SIRT1 activators also showed moderate protective effects on mitochondrial function in t-BHP cells by recovering oxygen consumption and increasing mitochondrial DNA content. Our results suggested that those compounds from TCMs attenuated oxidative stress-induced mitochondrial damage in cardiomyocytes through activation of SIRT1.

## 1. Introduction

Sirtuin type 1 (SIRT1) belongs to the family of class III histone deacetylases (HDAC) that consume NAD^+^ during deacylation cycle. It has been reported that, in mammals, SIRT1 plays a critical function in cellular metabolism and response to oxidative stress [1–4].

Recently, researchers have found that SIRT1 activators can protect mitochondrial function from oxidative-induced mitochondrial damage in various types of cell through regulating PGC-1*α* and multiple transcription factors [5–9], which are tightly related to mitochondrial biogenesis and metastasis [10, 11]. It is also reported that activators of SIRT1, such as resveratrol [12], can extend lifespan and regulate metabolic disorders [13–15]. Therefore, SIRT1 activators exhibit unique pharmacological potentials for treating mitochondrial dysfunction related diseases. Meanwhile, several clinical trials of SIRT1 activators such as SRT1720 for type 2 diabetes and obesity are under way [16].

Natural products have historically been regarded as an important resource of therapeutic agents in pharmaceutical discovery over the past century [17]. Traditional Chinese medicines (TCMs), as an important part of natural products, are mainly governed by empirical experience and fundamental theories such as the Yin and Yang concept [18]. TCMs with Qi Tonification effects including* Astragalus membranaceus* [19, 20],* Panax ginseng* [21, 22], and* Panax notoginseng* [23, 24] have been reported to exert protective effect against oxidative stress in mitochondria. Several compounds isolated from TCMs are reported to regulate SIRT1 activity [25]. However, a comprehensive screening of SIRT1 activators from TCMs has not yet been performed to investigate their protective effects on mitochondrial function against oxidative stress.

The aim of present study is to discover SIRT1 activators from TCMs and validate their activities against mitochondrial damage. A sensitive* in vitro* assay to screen SIRT1 activators was performed to discover bioactive compounds from TCMs, and the lead compounds were validated by liquid chromatography-mass spectrometry (LC-MS) analysis. Effects of identified SIRT1 activators on mitochondrial function were further investigated in cardiomyocytes exposed to tert-butyl hydroperoxide (t-BHP). ATP content, intracellular ROS formation, and activity of Mn-SOD were measured. Moreover, oxygen consumption and mitochondrial DNA content of cardiomyocytes were used to evaluate the effects of those SIRT1 activators on mitochondrial function.

## 2. Materials and Methods

### 2.1. Supplies and Chemicals

SIRT1 protein (human recombinant) and lysyl endopeptidase were purchased from Cayman Chemical (USA). Ginsenoside F1, ginsenoside Rc, and schisandrin A were purchased from Shanghai Winherb Medical Technology Company (China). Ginsenoside Rb_2_ was purchased from National Institute for Food and Drug control (Beijing, China).

### 2.2. Cell Culture

H9c2 (from Cell Bank of Chinese Science Academy, Shanghai, China) were cultured in DMEM (Corning, USA) containing 10% fetal bovine serum (Sigma, USA), 100 U/mL penicillin, and 100 *μ*g/mL streptomycin (Gibco, USA). All the cells were grown in 5% humidified CO_2_ atmosphere at 37°C.

### 2.3. Fluorescent Probe Based Assay for SIRT1 Modulation Effects of Compounds

The measurement of SIRT1 activity and effects of compounds on SIRT1 activation were performed by a previously reported fluorescent method [26]. Briefly, SIRT1 was incubated with a TPE-GK(Ac)YDD probe (20 *μ*M) in the presence of the tested compound (50 *μ*M). Fluorescence intensity was recorded by a TECAN infinite F200 microplate reader with excitation wavelength 320 nm and emission wavelength 465 nm.

A total of 195 constituents of TCMs were screened by the assay to evaluate their regulatory effects on SIRT1 activity. The detailed information related to the chemicals and their CAS number were illustrated in Supplementary Material available online at http://dx.doi.org/10.1155/2016/4206392. The inhibition or activation of SIRT1 was calculated by the following equation:

(1) $$\begin{array}{c} \text{Activation rate}\left(\;\%\;\right)=\left(\;\frac{\left(I_{s}-I_{s0}\right)/I_{s0}}{\left(I_{c}-I_{0}\right)/I_{0}}-1\;\right). \end{array}$$

*I*_*s*_ and *I*_*c*_ represented the fluorescence intensity of tested sample group with various test compounds and control group without test compounds. *I*_*s*0_ and *I*_0_ represented the fluorescence intensity of tested sample and control when incubated without SIRT1 protein. To explore the dose-related effects of these compounds, several active compounds, including ginsenoside Rb_2_, ginsenoside Rc, and schisandrin A, were further tested with the concentrations of 1, 10, 25, and 50 *μ*M.

### 2.4. Validation of SIRT1 Activators by LC-MS Analysis

In order to validate activation of SIRT1, SIRT1 (10 *μ*g/mL) was added to TPE-GK(Ac)YDD (20 *μ*M) and NAD^+^ (Sigma, 3 mM) for 1 h incubation at 37°C in the presence or absence of ginsenoside Rb_2_, ginsenoside F1, ginsenoside Rc, and schisandrin A (50 *μ*M), respectively. Samples were boiled at 100°C for 10 min to degenerate SIRT1 and terminate reaction.

Samples were analyzed by Agilent 1100 LC system (Agilent Technologies, USA) and Finnigan LCQ Deca XP^plus^ ion trap mass spectrometer with an ESI source (Thermo, USA). The acquisition parameters for LC/ESI-MS were as follows: nebulizing gas, high-purity nitrogen (N_2_); collision gas, high-purity helium (He); capillary voltage: −15 V; capillary temperature: 350°C; ion spray voltage: −3 kV; tube lens offset voltage: −30 V; mass range:* m/z* 100−1500. Chromatographic separation was performed by a reversed-phase ZORBAX SB-C_18_ analytical column. The mobile phase included water containing 0.1% (v/v) formic acid (A) and acetonitrile (B). The flow rate was 0.6 mL/min. A gradient program was carried out as the following profile: 0 min, 50% B; 5 min, 50% B; 30 min, 95% B; 40 min, 95% B.

### 2.5. Measurement of ATP Content and Intracellular ROS

H9c2 cells were seeded in 96 wells at the density of 4,000/well. The cells were preincubated with ginsenoside Rb_2_, ginsenoside F1, ginsenoside Rc, and schisandrin A (20 *μ*M) for 24 h before being exposed to t-BHP (300 *μ*M) for 1 h. Intracellular ATP content was measured by CellTiter-Glo^®^ Luminescent Assay kit (Promega) according to the instruction of manufacture. Intracellular ROS content was measured by DCFH-DA probe (5 *μ*M) whilst fluorescence intensity was recorded by a TECAN infinite F200 Multifunction microplate with excitation wavelength 485 nm and emission wavelength 535 nm. The changes of ATP content and ROS accumulation were calculated by comparing the luminescent or fluorescent signal of the treated cells with that of untreated H9c2 cells.

### 2.6. Detection of Mn-SOD Activity

Manganese superoxide dismutase (Mn-SOD) was an antioxidative enzyme, which protected against ROS-induced damage [27]. To measure Mn-SOD activity, H9c2 cells were seeded in 6-well plate in the density of 4 × 10^5^/mL. H9c2 cells were preprotected for 24 h by ginsenoside Rb_2_, ginsenoside F1, ginsenoside Rc, and schisandrin A (20 *μ*M) before being exposed to t-BHP (300 *μ*M) for 1 h. The cells were lysed and the concentration of total protein was measured by BCA assay kit. Mn-SOD activity in total protein was measured by Mn-SOD assay kit (Beyotime, China).

### 2.7. Oxygen Consumption Assay

2 × 10^6^ H9c2 cells were seeded in 100 mm culture plate. After being grown to stable attachment, cells were preincubated with ginsenoside Rb_2_, ginsenoside F1, ginsenoside Rc, and schisandrin A at final concentrations (20 *μ*M) for 18 h before being exposed to t-BHP (100 *μ*M) for 1 h. Cells were washed with PBS twice, subsequently collected by trypsinization followed by centrifugation, and resuspended in fresh medium. Respiratory activity was measured with a Clark-type oxygen electrode (Oxytherm, Hansatech Instruments, UK). An aliquot (1 mL) of suspended cells (2 × 10^6^ cells/mL) was placed in the air-tight liquid-phase oxygen electrode chamber. The system was maintained at 37°C. After equilibration, the slope of oxygen consumption in H9c2 cells was measured. Every 1 × 10^6^ cells oxygen consumption was calculated as the basic respiration rate of each group. The experiment was also performed in the presence of SIRT1 inhibitor, EX527 at final concentration of 20 *μ*M to investigate whether the effect of ginsenoside Rb_2_ can be prevented by SIRT1 inhibitor.

### 2.8. Measurement of Mitochondrial DNA Content

Real-time PCR was used to determine relative quantities of mitochondrial DNA content in H9c2 cells exposed to t-BHP and cells incubated with SIRT1 activators. Cells were preincubated for 24 h with ginsenoside Rb_2_, ginsenoside F1, ginsenoside Rc, and schisandrin A at final concentrations (20 *μ*M) before being exposed to t-BHP (300 *μ*M) for 1 h. Cells in normal condition were used as control group. Total DNA was extracted using Mammalian Genomic DNA Miniprep Kits (Sigma, USA). DNA was quantified by measuring *A*_260_ values, and 50 ng of total DNA was used for PCRs by GenElute™ QuantiFast SYBR Green PCR Kit (QIAGEN, Germany). Primers specific to the mitochondrial-encoded Atp6 gene (Fw: 5′-ATT ACG GCT CCT GCT CAT A-3′; Rev: 5′-TGG CTC AAC CAA CCT TCT A-3′) were used to assess mitochondrial DNA copy numbers. Primers designed against the nuclear-encoded Rpl13 gene (Fw: 5′-CAC AAG AAA ATG GCA CGC AC-3′; Rev: 5′-GAG CAG AAG GCT TCC TGG G-3′) were used for normalization. *C*_*T*_ values were obtained automatically. The number of mitochondrial genes was calculated by 2^−ΔΔ*C*_*T*_^ method.

### 2.9. Statistical Analysis

All values were expressed as the means ± SD. One way ANOVA was used to analyze differences among groups. Statistical analysis was performed using GraphPad Prism. *p* values of less than 0.05 were considered statistically significant.

## 3. Results

### 3.1. Screening of SIRT1 Activators from Compound Library of TCM

**Figure 1:**
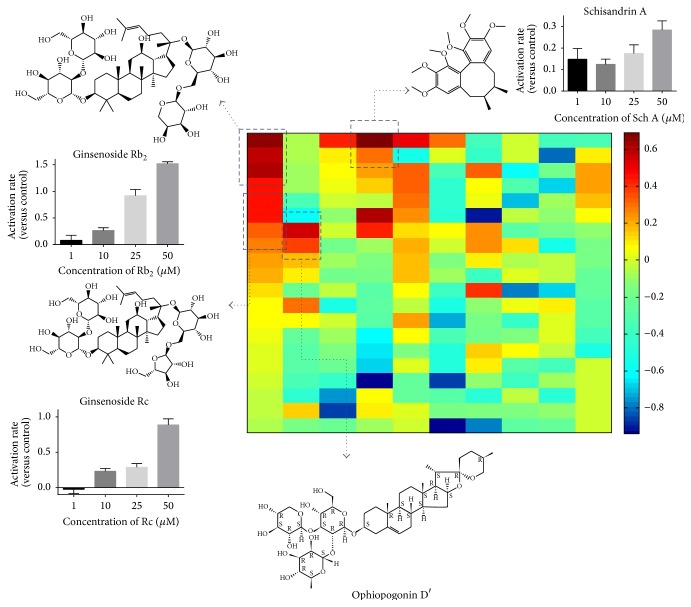
Screening results of 195 compounds from TCMs and dose dependent activation of representative activators.

To screen SIRT1 activators, we chose 195 compounds from TCMs with different efficacy, which were defined according to the TCMs theory. For example,* Panax ginseng* and* Ophiopogon japonicas* were regarded as Tonifying herbs, whilst* Schisandra chinensis* was Astringent herb and* Sophora flavescens* came from heat-clearing medicinal.  Figure 1 exhibited a heatmap of activation or inhibition rate of each compound. The corresponding values were listed in Supplementary Table. A total of 19 SIRT1 activators were found, including 20(S)-ginsenoside Rg_3_ (60%), ginsenoside Rb_3_ (28%), ginsenoside F1 (22%), and ginsenoside F2 (45%) from* Panax ginseng*, gypenoside XVII (43%) and notoginsenoside Ft1 (40%) from* Panax notoginseng*, polyphyllin I (24%), polyphyllin III (32%), polyphyllin VI (31%), and polyphyllin VII (32%) from* Paris polyphylla*, liriopesides B (65%) and* Liriope muscari* baily saponins C (57%) from* Liriope muscari*, ophiopogonin D′ (54%) from* Ophiopogon japonicas*, saikosaponin A (25%) from* Bupleurum chinense*, schisandrin B (30%)* from Schisandra chinensis*, and anisodine hydrobromide (60%) from* Anisodus tanguticus*. It was obvious that most of identified activators belonged to Tonifying herb. Moreover, potential inhibitors of SIRT1 almost belonged to herbs with heat-clearing efficacy.

Representative compounds were chosen to validate their activation effects on SIRT1 activity. Ginsenoside Rb_2_ showed 8% to 152% activation with the concentration range of 1~50 *μ*M. Ginsenoside Rc exerted 88% activation at the concentration of 50 *μ*M. Schisandrin A showed 28% activation at the concentration of 50 *μ*M.

### 3.2. Validation of SIRT1 Activators by LC-MS Analysis

**Figure 2:**
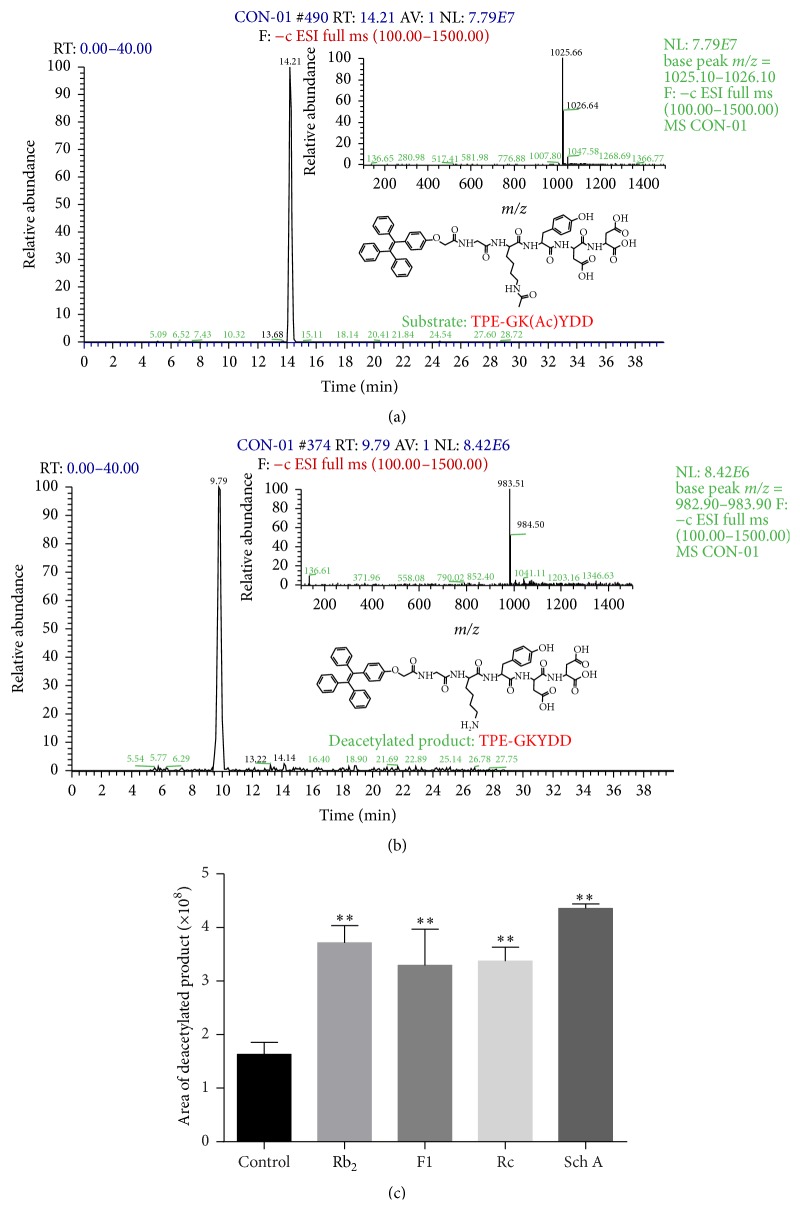
LC-MS results of SIRT1 activation. LC-MS chromatograms in negative ion mode and structures of (a) substrate TPE-GK(Ac)YDD and (b) deacetylated product TPE-GKYDD. (c) Area of deacetylated product in LC-MS chromatogram. SIRT1 was incubated with or without ginsenoside Rb_2_ (Rb_2_), ginsenoside F1 (F1), ginsenoside Rc (Rc), and schisandrin A (Sch A). Each bar represented the mean ± SD of triplicate experiments. Compared with control group, ^*∗∗*^ *p* < 0.01.

To validate the SIRT1-activation effect of the compounds, liquid chromatography-mass spectrometry (LC-MS) analysis was employed. The specific substrate of SIRT1, TPE-GK(Ac)YDD (Figure 2(a)), was incubated with SIRT1 (10 *μ*g/mL) and NAD^+^ (3 mM) for 1 h. The reaction product was identified by LC-MS based on the molecular weight. As shown in Figure 2(b), the deacetylated peptide, TPE-GKYDD, was detected with a loss of Ac (43 Da). When analyzing the deacetylated reaction in the presence of SIRT1 activators, including ginsenoside Rb_2_, ginsenoside F1, ginsenoside Rc, and schisandrin A, the peaks of deacetylated product were significantly elevated (Figure 2(c)). Our findings indicated that these compounds activated SIRT1 in enzymatic reaction.

### 3.3. Effects of SIRT1 Activators on Cardiomyocytes Oxygen Consumption

**Figure 3:**
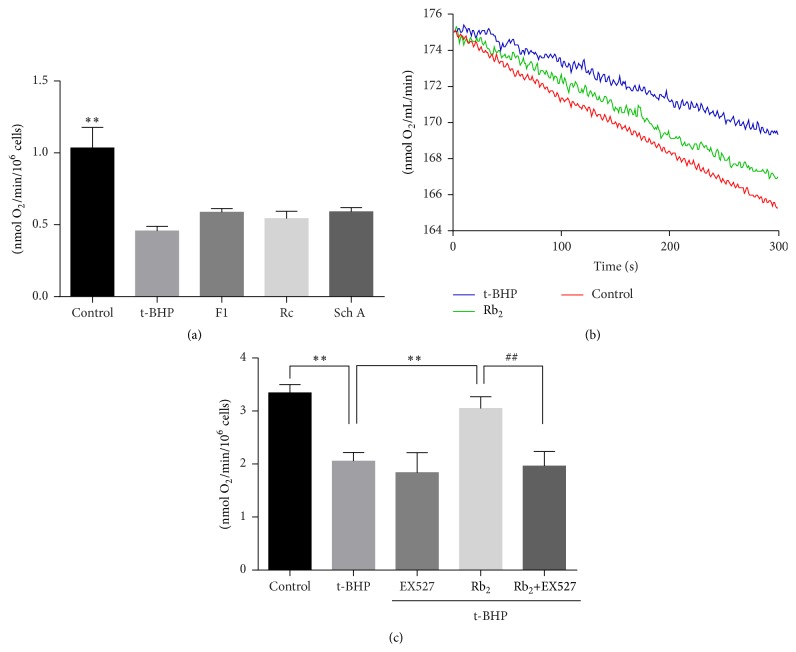
Effects of SIRT1 activators on mitochondrial oxygen consumption. (a) Respiration rates of H9c2 cells, t-BHP injured cells, and t-BHP injured cells preincubated with F1, Rc, and Sch A. (b) Representative curves of oxygen consumption recorded by the Clark-type oxygen electrode, and t-BHP injured cells were preincubated with Rb_2_. (c) In the presence of EX527, the effect of ginsenoside Rb_2 _was blocked. Each bar represented the mean ± SD of triplicate experiments. Compared with t-BHP group, ^*∗∗*^ *p* < 0.01. Compared with t-BHP + Rb_2_ + EX527 group, ^##^ *p* < 0.01.

The effects of SIRT1 activators on mitochondrial function were further investigated by measuring cellular respiration in H9c2 cells. As shown in Figure 3(a), basal respiration of t-BHP treated cardiomyocytes was significantly dropped comparing with control group. Preincubation with ginsenoside Rb_2_, ginsenoside F1, ginsenoside Rc, and schisandrin A attenuated the decrease of oxygen consumption.  Figure 3(b) showed the representative oxygen consumption slope of normal cells (1.65 ± 0.31 nmol O_2_/mL/min), t-BHP treated cells (1.10 ± 0.25 nmol O_2_/mL/min), and ginsenoside Rb_2_ treated cells (1.43 ± 0.25 nmol O_2_/mL/min). The results suggested that these SIRT1 activators recovered the oxygen consumption rate in t-BHP injured cardiomyocytes. In the presence of SIRT1 inhibitor EX527 [28, 29], the protective effect of ginsenoside Rb_2_ was blocked, which indicated that the effect of ginsenoside Rb_2_ to recover the oxygen consumption rate was SIRT1 dependence (Figure 3(c)).

### 3.4. Effects of SIRT1 Activators on ATP Content and ROS Accumulation

**Figure 4:**
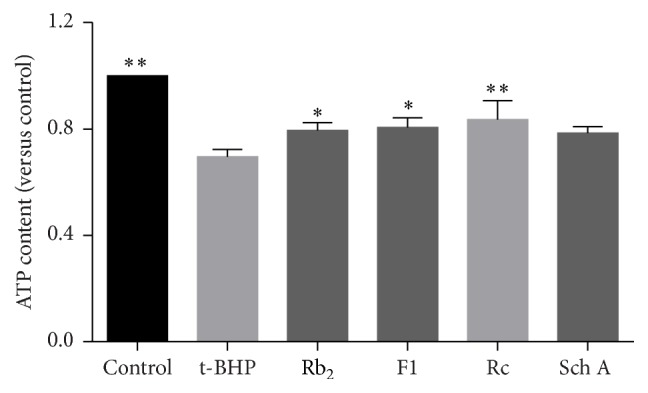
Effects of SIRT1 activators on the ATP content in t-BHP treated H9c2 cells. Each bar represented the mean ± SD of triplicate experiments. Compared with t-BHP group, ^*∗*^ *p* < 0.05 and ^*∗∗*^ *p* < 0.01.

As a specific parameter of mitochondrial function, intracellular ATP contents in cardiomyocytes exposed to oxidative stress were measured. After cells were exposed to t-BHP for 1 h, the content of ATP was significantly decreased. As shown in Figure 4, preprotection of cells by ginsenoside Rb_2_, ginsenoside F1, ginsenoside Rc, and schisandrin A led to the recovery of ATP content, suggesting that those SIRT1 activators reversed the decreased mitochondrial energy metabolism induced by t-BHP.

**Figure 5:**
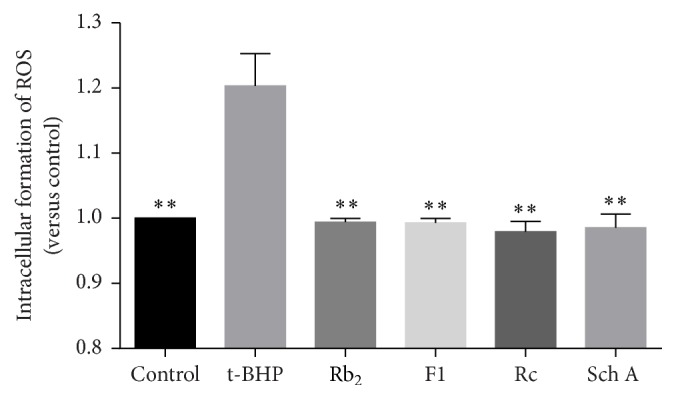
Effects of SIRT1 activators on the intracellular ROS level. Each bar represented the mean ± SD of triplicate experiments. Compared with t-BHP group, ^*∗∗*^ *p* < 0.01.

Mitochondrial oxidative stress was often caused by increased intracellular ROS formation.  Figure 5 showed that the intracellular ROS was significantly increased after t-BHP treatment. Pretreatments of SIRT1 activators, ginsenoside Rb_2_, ginsenoside F1, ginsenoside Rc, and schisandrin A, kept intracellular ROS levels on the normal condition.

### 3.5. Effects of SIRT1 Activators on Mn-SOD Activity

**Figure 6:**
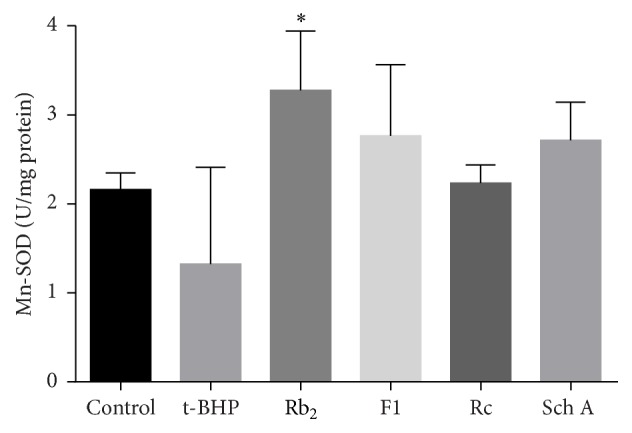
Effects of SIRT1 activators on Mn-SOD activity. Each bar represented the mean ± SD of triplicate experiments. Compared with t-BHP group, ^*∗*^ *p* < 0.05.

Mn-SOD was one of the antioxidative enzymes in mitochondria that assured mitochondrial oxidative stress resistance. As shown in Figure 6, when cells were exposed to t-BHP (300 *μ*M) for 1 h, activity of Mn-SOD was decreased. Preprotection of cells by ginsenoside Rb_2_, ginsenoside F1, ginsenoside Rc, and schisandrin A enhanced the activity of Mn-SOD compared with t-BHP group.

### 3.6. Effects of SIRT1 Activators on Mitochondrial DNA Content

**Figure 7:**
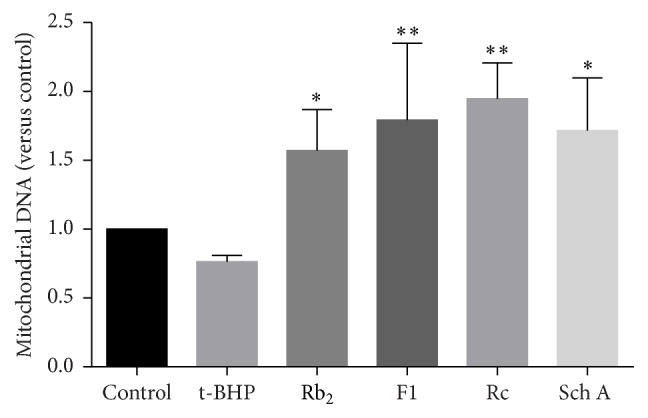
Effects of SIRT1 activators on mitochondrial DNA content. Each bar represented the mean ± SD of triplicate experiments. Compared with t-BHP group, ^*∗*^ *p* < 0.05 and ^*∗∗*^ *p* < 0.01.

To verify the effect of ginsenoside Rb_2_, ginsenoside F1, ginsenoside Rc, and schisandrin A of mitochondrial protection or biogenesis, cells were injured by t-BHP after compounds preprotection, and then mitochondrial DNA content was analyzed. As shown in Figure 7, t-BHP treatment reduced mitochondrial DNA content compared to control group. Pretreatments of ginsenoside Rb_2_, ginsenoside F1, ginsenoside Rc, or schisandrin A significantly elevated the mitochondrial DNA content. Our findings suggested that these natural SIRT1 activators facilitated mitochondrial biogenesis.

## 4. Discussion

In the present study, we screened and identified 19 SIRT1 activators, such as ginsenoside Rg_3_, ginsenoside Rb_2_, ginsenoside Rb_3_, ginsenoside F1, and ginsenoside Rc from* Panax ginseng*, ophiopogonin D′ from* Ophiopogon japonicas,* and schisandrin A and schisandrin B from* Schisandra chinensis*. Interestingly, those herbs consisted of a traditional Chinese formula named Shengmai San, which have been clinically used for the treatment of coronary heart diseases [30, 31] and heart failure [32, 33]. Four SIRT1 activators from Shengmai San, including ginsenoside Rb_2_, ginsenoside F1, ginsenoside Rc, and schisandrin A, were validated by LC-MS analysis and we found their effects against mitochondrial oxidative damage in further study. Our findings were in accordance with previous reports on other cell lines. Ginsenoside Rc was reported to suppress oxidative stress in HEK293T cells [34], whilst schisandrin A inhibited apoptosis induced by H_2_O_2_ in intestinal epithelial cells [35].

Mitochondrial dysfunction has been one of mechanisms in organ injuries and diseases due to its influence on ATP formation, metabolism, and apoptosis [36]. Our findings indicated that those SIRT1 activators elevated ATP content, prevented ROS formation, and increased the activity of Mn-SOD. Mitochondrial DNA content and oxygen consumption were also moderated by SIRT1 activators. Those results indicated that SIRT1 activators protected mitochondrial function by improving mitochondrial DNA content, which led to promotion of ATP content, mitochondrial oxygen consumption, and reduction of ROS formation.

## 5. Conclusion

In summary, we identified 19 SIRT1 activators from TCMs. Four active compounds, ginsenoside Rb_2_, ginsenoside F1, ginsenoside Rc, and schisandrin A, exerted significant activities against t-BHP induced oxidative damage in cardiomyocytes. Our findings provided useful evidence to illustrate the cardioprotective effects of TCMs with Tonification effects and led to a new insight into the scientific illustration of TCMs theory.

## Supplementary Material

A total of 195 constituents of TCMs were screened by the assay to evaluate their regulatory effects on SIRT1 activity. The Supplementary Material illustrated the detailed information related to the chemicals and their CAS number. And the Supplementary Material also provided the orders, activation/inhibition rates and function of 195 constituents in the heatmap of Figure 1.

## Abbreviations

TCMs: Traditional Chinese medicines
AIE: Aggregation induced emission
LC-MS: Liquid chromatography-mass spectrometry
t-BHP: tert-Butyl hydroperoxide
ROS: Reactive oxygen species
F1: Ginsenoside F1
Rc: Ginsenoside Rc
Sch A: Schisandrin A
Rb_2_: Ginsenoside Rb_2_.
